# Waveform Selectivity at the Same Frequency

**DOI:** 10.1038/srep09639

**Published:** 2015-04-13

**Authors:** Hiroki Wakatsuchi, Daisuke Anzai, Jeremiah J. Rushton, Fei Gao, Sanghoon Kim, Daniel F. Sievenpiper

**Affiliations:** 1 Center for Innovative Young Researchers, Nagoya Institute of Technology, Gokiso-cho, Showa, Nagoya, Aichi, 466-8555, Japan; 2 Department of Electrical and Electronic Engineering, Nagoya Institute of Technology, Gokiso-cho, Showa, Nagoya, Aichi, 466-8555, Japan; 3 Applied Electromagnetics Group, Electrical and Computer Engineering Department, University of California, San Diego, 9500 Gilman Drive, La Jolla, CA 92093, USA; 4 The Science and Technology on Antenna and Microwave Laboratory, Xidian University, Xi'an, Shaanxi 710071, China

## Abstract

Electromagnetic properties depend on the composition of materials, i.e. either angstrom scales of molecules or, for metamaterials, subwavelength periodic structures. Each material behaves differently in accordance with the frequency of an incoming electromagnetic wave due to the frequency dispersion or the resonance of the periodic structures. This indicates that if the frequency is fixed, the material always responds in the same manner unless it has nonlinearity. However, such nonlinearity is controlled by the magnitude of the incoming wave or other bias. Therefore, it is difficult to distinguish different incoming waves at the same frequency. Here we present a new concept of circuit-based metasurfaces to selectively absorb or transmit specific types of waveforms even at the same frequency. The metasurfaces, integrated with schottky diodes as well as either capacitors or inductors, selectively absorb short or long pulses, respectively. The two types of circuit elements are then combined to absorb or transmit specific waveforms in between. This waveform selectivity gives us another degree of freedom to control electromagnetic waves in various fields including wireless communications, as our simulation reveals that the metasurfaces are capable of varying bit error rates in response to different waveforms.

The advent of new material properties has led to the development of new applications in electromagnetism. For example, metamaterials^1^,^2^,^3^, composed of sub-wavelength resonant structures, readily enable us to use negative- or zero-refractive indices^2^ as well as artificially engineered high impedance surfaces^3^ which allow surface wave properties to be controlled. These unusual properties were applied to the development of a diffraction-limitless lens^4^,^5^, cloaking device^6^,^7^,^8^, unusually thin absorbers (<*λ*/4 where *λ* is the wavelength of the incoming wave)^9^,^10^,^11^, etc. Importantly, such new electromagnetic properties and applications were widely exploited in other disciplines such as acoustics^12^,^13^,^14^,^15^, thermodynamics^16^ and vibration engineering^17^,^18^. Moreover, a recent study on metasurface absorbers containing circuit elements such as diodes and capacitors demonstrated a new property called *waveform dependence*^19^,^20^. Interestingly these metasurfaces absorbed only short sine wave pulses, while transmitting continuous waves (CWs) even at the same frequency. In this study we present a new concept of metasurfaces to fully control waveforms, i.e. selective absorption of either short pulses or CWs as well as absorption or transmission of specific waveforms in between. This new property termed *waveform selectivity* allows us to distinguish incoming waves in an unusual manner depending not only on the frequency but also on the waveform. Thus the waveform selectivity has a potential to develop new kinds of techniques and applications in electromagnetism as well as in other disciplines.

The waveform selectivity is made possible by integrating the rectification of microwave diodes with the time-domain responses of capacitors and inductors (Figs. 1(a) and 1(b)). First, a diode converts the frequency of an incoming signal to an infinite set of frequency components, but most of the energy is at zero frequency^10^. The conversion to zero frequency is further enhanced if a four-diode bridge is used^19^ (see Supplementary Information for details). Also, a capacitor has an impedance calculated from 1/*jωC*, where *j*^2^ = −1, *ω* is the angular frequency (*ω* = 2*πf* and *f* is the frequency) and *C* is the capacitance. Since the rectified signal contains the zero frequency component, the capacitor stores the energy but is gradually charged up. This indicates that capacitors allow current to come in *during* an initial time period. On the other hand, an inductor has impedance of *jωL*, where *L* is the inductance, and generates an electromotive force opposing the incoming current. This force, however, is gradually weakened due to the zero frequency component, and more current comes in. Thus, inductors accept incoming current *after* an initial time period.

In addition, integration of these time domain responses with a resistor leads to effective absorption of either short or long waveforms. Fig. 1(c) illustrates such circuit configurations deployed between square patches of metasurfaces. In this figure a capacitor is connected to a resistor in parallel, or an inductor in series. Incoming waves have electric field normal to the metasurfaces, which charges the edge of each patch either positively or negatively and thus generates strong electric field across the gaps (this electric field turns on the diodes). Under these circumstances the capacitor is capable of fully storing the energy of a short pulse during the illumination, and discharges it into the resistor, which dissipates the energy before a next short pulse comes in. For a CW, however, the capacitor is fully charged up. As a result, the incoming wave transmits over the metasurface. Regarding the inductor, a short pulse cannot be rectified by diodes due to the presence of the electromotive force, resulting in no energy dissipation in the series resistor. However, reduction of the force due to long-term illumination permits the rectification, leading to energy dissipation in the resistor. For these reasons a short pulse or CW is expected to be absorbed by the combination of a resistor with a capacitor or inductor, respectively.

Importantly, selective absorption and transmission demonstrated below are not due to the variation in the bandwidth of the incoming frequency, since the bandwidth of the signals is small compared to that of the surface without any nonlinear elements (e.g. provided that frequency and pulse width are respectively 4.0 GHz and 50 ns as set below, the one cycle is 0.25 ns and corresponds to only a 200th of the pulse duration).

On the basis of this theory, a metasurface was designed and built up as in Figs. 1(d) and 1(e) in order to absorb incoming surface waves. The simulation model had nine periodic unit cells along the propagation direction of a surface wave on the bottom of a TEM (transverse electromagnetic) waveguide. The measurement sample was fabricated under the same circumstances but with a few differences. For example, since the measurement required to use TE (transverse electric) waveguides (WR284 for up to 3.95 GHz and WR187 beyond 3.95 GHz) as realistic waveguides, the measurement sample had several unit cells along not only the direction of the wave propagation (***k*** in Fig. 1(e)) but also that of the incident magnetic field (***H***) to fully occupy the bottoms of the waveguides. In addition, the measurement used commercial schottky diodes (Avago; High Frequency Detector Diodes HSMS-2863/2864), while the simulation used a SPICE model. The capacitors and inductors respectively had capacitance *C* = 1 nF and inductance *L* = 100 *μ*H, while the resistors used with the capacitors and inductors respectively had resistances *R_c_* = 10 kΩ and *R_l_* = 5.5 Ω, where the measurement sample of the inductor-based metasurface did not use resistor chips, since the inductor chips already contained some resistance value, which was the same as *R_l_*. The self-resonant frequencies of the capacitor and inductor chips, *f_c_* and *f_l_*, were respectively 300 and 10 MHz. Note that the time constants of the capacitor-based and inductor-based metasurfaces, determined by *R_c_C* and *L*/*R_l_*, were respectively 10 *μ*s and ~18 *μ*s. More details on the simulation and measurement are described in Fig. 1 and Supplementary Information.

## Results

**Figure 2:**
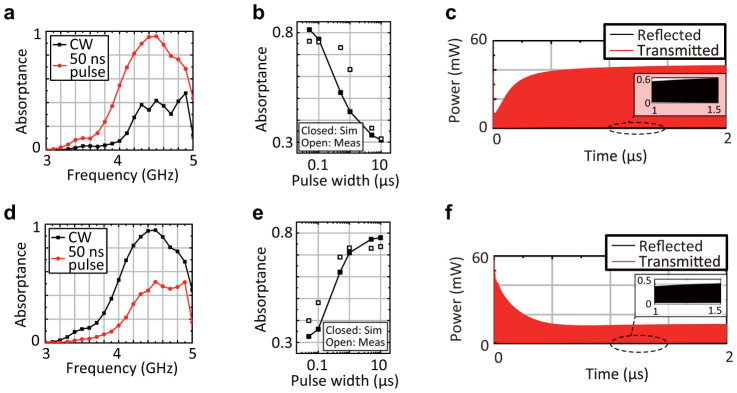
Absorbing performance of the capacitor-based and inductor-based metasurfaces. (a) and (d) The capacitor- and inductor-based metasurfaces containing the circuit configurations of Fig. 1 (c) numerically exhibited stronger absorption for a short pulse (50 ns long) and CW, respectively. (b) and (e) At 4.2 GHz each of the capacitor-and inductor-based metasurfaces numerically showed a clear transition between the short pulse and CW, as plotted by the closed squares of (b) and (e), respectively. Similar trends were experimentally demonstrated at 4.0 GHz (the open squares). (c) and (f) The time domain responses of these metasurfaces are seen in (c) and (f), respectively.

First of all, a capacitor-based metasurface and inductor-based metasurface were numerically tested with 15 dBm signals at different frequencies as shown in Figs. 2(a) and 2(d), respectively. As a result, the capacitor-based and inductor-based metasurfaces respectively absorbed short pulses (50 ns long) and CWs more effectively. Next, the frequency was fixed at 4.2 GHz, where either a short pulse or CW was strongly absorbed, while the other was weakly absorbed. Under this circumstance, the capacitor-based metasurface demonstrated a clear transition between the short pulse and CW as plotted by the closed squares of Fig. 2(b)^19^. This is because the capacitors used were gradually charged up, as the pulse width increased. The measurement was performed under the same circumstances except the input frequency set to 4.0 GHz due to a minor shift of the entire feature to a lower frequency region (see Fig. 11 of Supplementary Information). As plotted by the open squares of Fig. 2(b), the measurement result also demonstrated that the absorbing performance decreased, as the pulse width increased. On the other hand, the inductor-based metasurface gradually enhanced the absorbing performance by increasing the pulse width as Fig. 2(e), since the electromotive force was weakened and the incoming rectified energy was dissipated with the series resistors. The reasons for the differences between these simulated and measured results are explained by circuit parasitics (e.g. extra capacitances in diodes) and superimposed direct current characteristics. The former reason increased the time constant of the capacitor-based metasurface, while the latter decreased that of the inductor-based metasurface. The time domain responses of these two waveform-dependent metasurfaces to 15 dBm CWs are seen in Figs. 2(c) and 2(f), respectively. As expected from Figs. 2(b) and 2(e), the capacitor-based (inductor-based) metasurface gradually increased (decreased) the transmitted power. These plots also show that the reected powers were limited.

**Figure 3:**
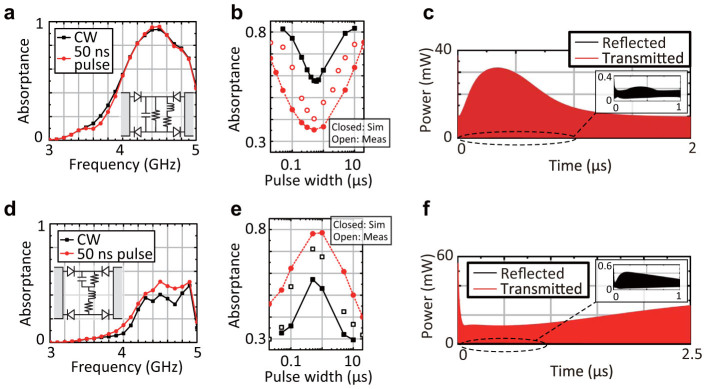
Selective transmission and absorption achieved through use of both capacitors and inductors. (a) and (d) The insets illustrate the metasurfaces combining both circuit configurations, respectively, (a) in parallel and (d) in series. The parallel-type metasurface numerically shows strong absorption for both of a short pulse and CW, while the series-type shows limited absorption for both of them. (b) The parallel-type metasurface selectively transmits the waveforms which were weakly absorbed by both of the individual capacitor-based and inductor-based metasurfaces in Fig. 2 (see the closed squares in Fig. 3(b)). The variation of the absorptance can be controlled by modifying the time constants of the individual metasurfaces, i.e. *R_c_C* and *L*/*R_l_* (the closed circles). More specifically the black curve used (*C*, *L*, *R_c_*, *R_l_*) = (1 nF, 100 *μ*H, 10 kΩ, 5.5 Ω), while the red curve used (*C*, *L*, *R_c_*, *R_l_*) = (100 pF, 1 mH, 10 kΩ, 31.2 Ω). The measurement result supports the feasibility of the numerical simulation as well (the open circles). (c) The time domain response of the parallel-type metasurface (the closed squares of Fig. 3(b)) is plotted. (e) In contrast, the series-type metasurface absorbs the waveforms which were strongly absorbed by both of the individual metasurfaces in Fig. 2 (see the closed squares in Fig. 3(e)). Despite difference due to circuit parasitics and superimposed direct current characteristics, such a waveform selectivity is experimentally realisable (the open squares). Similarly with the parallel type, the variation of the absorptance can be controlled by the time constants (the circles). More specifically the black curve used the same values as the black curve of Fig. 3(b), while the red curve used (*C*, *L*, *R_c_*, *R_l_*) = (100 pF, 1 mH, 10 kΩ, 31.2 Ω).(f) The time domain response of the series-type metasurface (the circles of Fig. 3(e)) is plotted.

Such a waveform dependence can be more exibly designed by combining each of the circuit configurations with another. The insets of Figs. 3(a) and 3(d) illustrate circuit configurations containing the two types of circuit elements either in parallel or in series. For example, previously the individual capacitor-based metasurface absorbed short pulses but transmitted long pulses, which can be now absorbed by the parallel inductor part as demonstrated in Fig. 3(a). Interestingly, however, such a metasurface can still transmit some waveforms that were weakly absorbed by both of the individual metasurfaces (the closed squares of Fig. 3(b)). In this case, the absorptance value at each pulse width is close to the *larger* value of the individual structures (cf. Figs. 2(b) and 2(e)). Besides, as plotted by the closed circles of Fig. 3(b), the variation of the absorptance curve can be controlled by varying the time constants *R_c_C* and *L*/*R_l_* (now *C* = 100 pF, *L* = 1 mH, *R_c_* = 10 kΩ, *R_l_* = 31.2 Ω, *f_c_* = 1.02 GHz and *f_l_* = 2.4 MHz), since these parameters determine the saturation of each curve. This was experimentally demonstrated by the open circles of Fig. 3(b), where an incoming wave was more transmitted when the pulse width was around 0.5 *μ*s. The differences between the measured and simulated results were mainly due to some circuit parasitics and the superimposed direct current characteristics as explained above. Such behaviour of the parallel-type metasurface (the closed squares of Fig. 3(b)) can also be understood from Fig. 3(c), which reveals temporal enhancement of transmitted power.

In contrast, the series-type metasurface drawn by the inset of Fig. 3(d) can selectively absorb specific waveforms. Again, in Fig. 2 the individual capacitor-based metasurface temporarily stored the incoming energy in capacitors to dissipate it in resistors. However, this is now prevented by the electromotive force of the inductors. Likewise, long-pulse current cannot be absorbed by the inductor part, as the capacitors are fully charged up before the current reaches the inductor part. For these reasons, both a short pulse and CW are weakly absorbed as simulated in Fig. 3(d). However, the series-type metasurface still absorbs some waveforms that were strongly absorbed by both of the individual metasurfaces. This is demonstrated by the closed squares of Fig. 3(e), which combined the circuit configurations used in Figs. 2(b) and 2(e). Unlike the parallel-type metasurfaces, the absorptance value of the series-type metasurface at each pulse width is close to the *smaller* value of the individual structures (cf. Figs. 2(b) and 2(e)). This waveform selectivity is experimentally realisable as plotted by the open squares of Fig. 3(e), although circuit parasitics and superimposed direct current characteristics caused some difference. Similarly with the parallel case, this variation can be further increased by changing the time constants as seen from the circles of Fig. 3(e) (now *C* = 10 nF, *L* = 10 *μ*H, *R_c_* = 10 kΩ, *R_l_* = 2 Ω, *f_c_* = 57.3 MHz and *f_l_* = 45 MHz). The time domain response of such a series-type metasurface (the circles of Fig. 3(e)) is plotted in Fig. 3(f), which also supports temporal reduction of the transmitted power.

**Figure 4:**
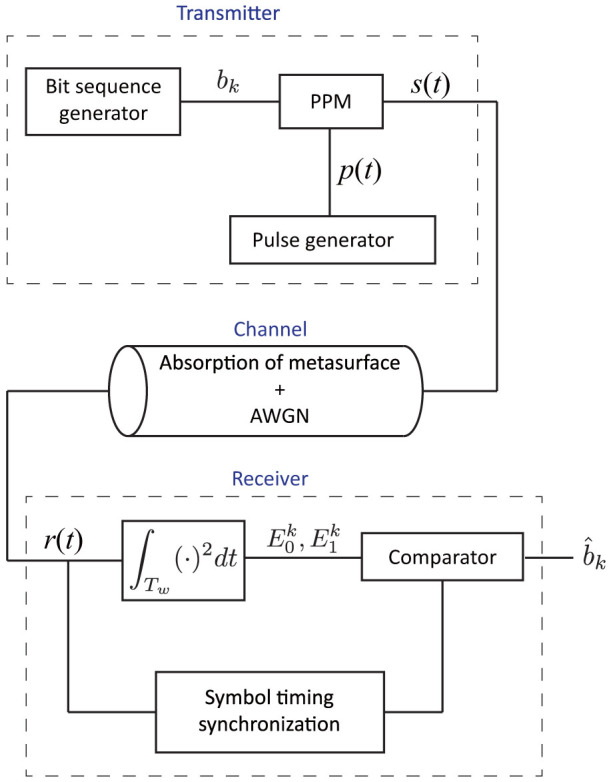
Simulation overview of wireless communication performance evaluation.

These waveform selectivities are expected to develop new kinds of techniques and applications in electromagnetics, especially in wireless communications as demonstrated below. Here the structure of the transmitter is shown in Fig. 4. The transmitter uses a binary pulse position modulation (PPM) scheme, in which bit information is sent as two pulse positions. The block of the PPM controls a programmable time delay, which determines when the pulse generator will be triggered. Assuming *K* as the total number of transmitted bits, the PPM signal *s*(*t*) can be expressed as
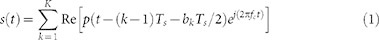
where *b_k_* is the *k*-th transmitted bit information, namely, 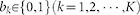, and *T_s_* is the symbol duration. Here, *p*(*t*) represents the baseband signal, which is given by
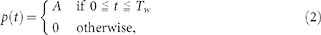
where *A* and *T_w_* are the amplitude of the baseband signal and the pulse width, respectively. Note that, in this paper, the bandwidth of the baseband signal *f_b_* = 1/*T_w_* is much smaller than the carrier frequency *f_c_* of 4.4 GHz (narrow-band condition). From the experimental results in Fig. 3, we observed that the metasurfaces worked well with pulse widths longer than 0.01 *μ*s. This means that the maximum bandwidth limit reaches only 100 MHz. Here this bandwidth is just about 2% of the carrier frequency used in the communication system. Therefore, even if we choose the maximum bandwidth of 100 MHz, the channel still satisfies the narrow-band condition. Consequently, even if we set several kinds of the pulse width (we set *T_w_* to 20, 2000 and 20000 ns in this paper), the transmitted signal can be assumed as if it has only a single frequency component of the carrier frequency, namely, the three channels with 20-, 2000- and 20000-*μ*s-long pulse widths can be assumed to have the same channel conditions. In addition, we use the same pulse interval for different pulse widths in order to fix the data speed at the same value.

Fig. 4 also shows the structure of the receiver. Here, we pay attention to the energy detection (non-coherent detection) as the received detection scheme. In the energy detection, since the binary PPM scheme chooses one from two location assignments in the *k*-th symbol, we calculate two kinds of *k*-th energies for the corresponding pulse locations from the received signal *r*(*t*) at the two kinds of time durations as follows:
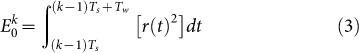



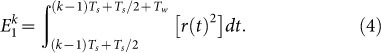
Comparing 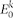 with 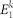, the received bit information 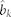 can be decided as
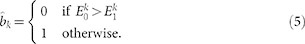
As seen from this equation, the PPM requires no threshold. On the other hand, on-off keying (OOK) modulation, which is often used in digital communications with a pulse modulation scheme, requires a threshold to distinguish signals and noise. Because the threshold depends on the signal-to-noise power ratio (SNR), it is difficult to determine it in advance. In this sense, the PPM is superior to the OOK modulation. Furthermore, the symbol timing in Fig. 4 is synchronised with pilot signals.

**Figure 5:**
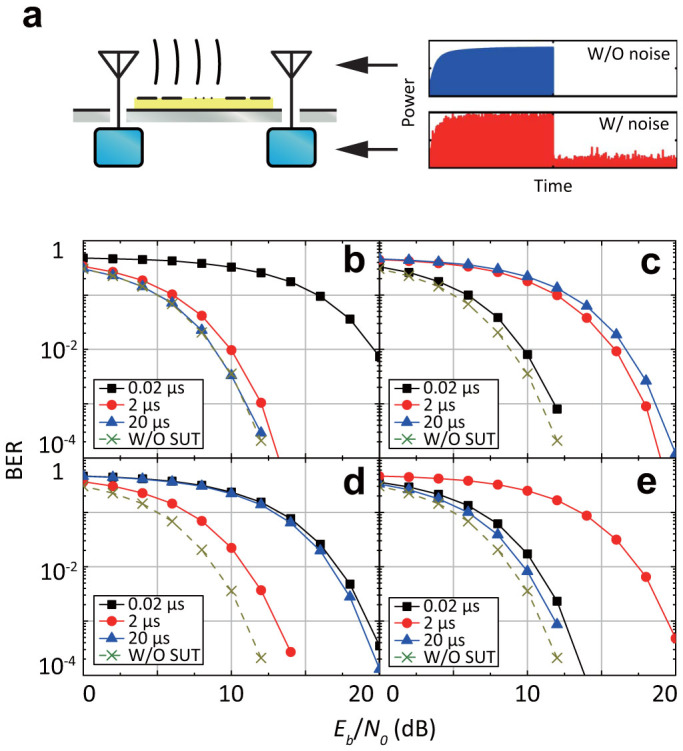
Waveform-selective wireless communications. (a) The overview of a wireless communication simulation where a surface wave propagates above metasurfaces. The signal received by an antenna (the blue line) (assumed as transmitted powers in TEM waveguide simulations) experiences the AWGN channel inside the demodulating circuit (the red line). (b)–(e) The BER performances for a simple narrow-band modulation, e.g. a PPM scheme, as a function of the energy per bit to the noise power spectral density ratio (*E_b_*/*N*_0_) in the communication system equipped with the capacitor-based, inductor-based, parallel-type and series-type metasurfaces, respectively. Optimal pulse widths for the best BER performances are determined by the waveform selectivity of the metasurfaces.

Fig. 5 shows bit error rate (BER) performances using four-types of waveform-selective metasurfaces (Figs. 2(b) and (e) and the closed circles of Figs. 3(b) and (e), respectively, for Figs. 5(b), (c), (d) and (e)) together with transmitted powers calculated from TEM waveguide simulations as the received signals (i.e. as the blue line of Fig. 5(a)). These signals then experience the additive white Gaussian noise (AWGN) channel inside the demodulator (the red line). In Figs. 5(b) to (d) a signal becomes more erroneous due to the selective absorption of the metasurface, if the curve is shifted upwards from that without surface under test (SUT). For example, in Fig. 5(b), the 20-*μ*s-long signal exhibited almost the same result as that without SUT, whereas the 0.02-*μ*s-long signal showed reduced performance due to the waveform-selective metasurface. That is, in this case, the proposed waveform-selective communication can receive the signal with 20 *μ*s pulse width and eliminate that with 0.02 *μ*s pulse width. Note that even in these simulations our metasurfaces are not used as a band pass filter to eliminate low or high frequency components. From this perspective, it can be concluded that the waveform-selective wireless communication successfully receives wireless signals with arbitrary pulse widths even at the same carrier frequency.

Importantly, we employed no other communications techniques, such as the code division multiple access (CDMA) and orthogonal frequency division multiple access (OFDMA) techniques^21^,^22^, which basically make use of wideband spectrum characteristics. In other words, we just considered a simple narrow-band modulation technique. Nevertheless, the waveform-selective communication can distinguish the narrow-band signals even at the same carrier frequency of 4.4 GHz by adapting several kinds of pulse widths. Moreover, the waveform selectivity can be combined with other wireless communication techniques including the multiple access techniques such as CDMA and OFDMA. This idea can potentially solve a long-lasting problem in available radio frequency resources, which are limited by a growing demand on wireless communications^23^, since the wave-form selectivity allows us to effectively share even the same frequency resource by assigning different pulse widths.

## Discussion

**Figure 6:**
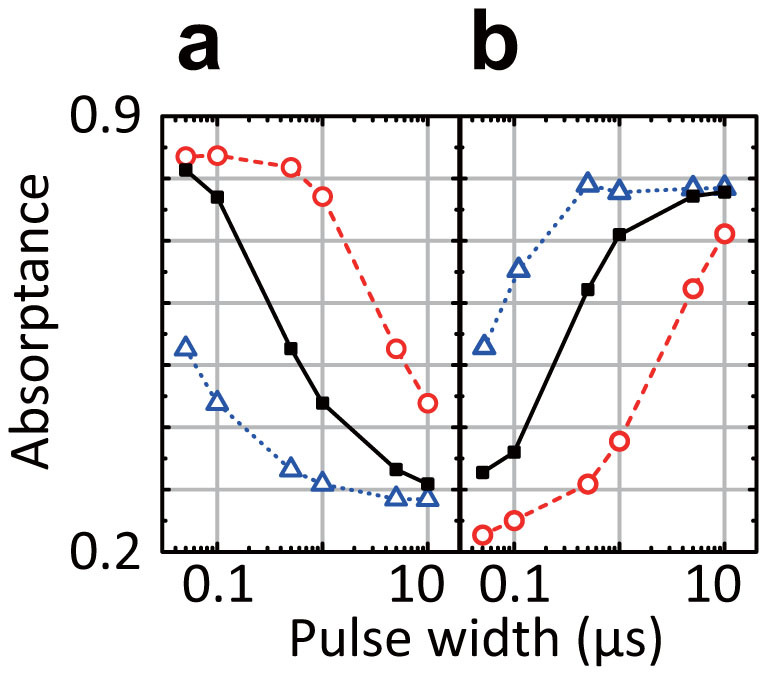
Pulse width dependences of (a) a capacitor-based metasurface with various capacitances and (b) an inductor-based metasurfaces with various inductances. These metasurfaces used the same conditions as those of Figs. 2(b) and 2(e) except for the capacitance and inductance. The results using the default capacitance and inductance values are plotted with the solid curves. These values are respectively increased and decreased by a factor of 10 in the dashed curves and dotted curves.

The time constants are very important to determine how the waveform-dependent metasurfaces behave in response to the pulse width of an incoming wave. This is demonstrated in Figs. 6(a) and 6(b), where the time constants of the metasurfaces used in Figs. 2(b) and 2(e) were varied, respectively. The dashed lines increased the default capacitance or inductance (the solid) by a factor of 10, while the dotted lines decreased the default values by a factor of 10. For simplicity, in these simulations the only value changed was either the capacitance or inductance, although realistically these changes have inuence over the self-resonant frequencies. As a result, it turned out that increasing the capacitance and inductance (i.e. increasing the time constants *R_c_C* and *L*/*R_l_*) led to shifting the original curves (the solid) to the right, since these changes made the capacitors store more incoming energy and the inductors maintain the electromotive force longer. On the other hand, the reductions of the capacitance and inductance resulted in shifting the original curves to the left. This is because the capacitors store less energy and the inductors maintain the electromotive force shorter. It is very important to understand from these figures that these pulse width dependences are readily controllable by changing the time constants only, as long as the necessary time constant values (i.e. the actual circuit components) are available. Therefore, the characteristics of the waveform selectivity are independent of the interaction between periodic unit cells, (here primarily capacitive coupling in gaps), although this interaction determines the resonant frequency around which the waveform selectivity is achieved.

## Conclusion

In summary we have demonstrated a new concept of metasurfaces, which selectively absorb or transmit specific waveforms even at the same frequency. Similarly with other metasurfaces^24^,^25^, these waveform-selective metasurfaces can be deployed not only as a coating on ordinary conducting surfaces but also as part of devices, which gives them additional functionality to control electromagnetic waves. For example, integrating these metasurfaces with antennas leads to sensing specific signals, as our simulation demonstrated that they can vary BER performances in accordance with pulse widths.

## Author Contributions

H.W. and D.F.S. jointly conceived of the entire idea to integrate the rectification into metasurfaces together with the time-domain responses of capacitors and inductors. H.W. designed, simulated and measured the metasurfaces. S.K. supported part of the measurement. D.A. performed simulations for wireless communications. All the authors discussed the results. H.W. and D.A. wrote the manuscript and D.F.S. revised it. S.K., F.G. and J.J.R. also contributed to the revision.

## Supplementary Material

Supplementary Information Supplementary Information

## Figures and Tables

**Figure 1:**
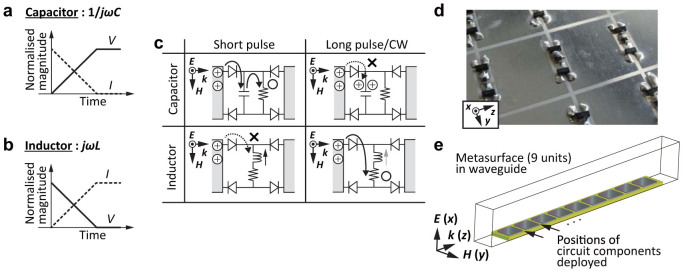
Fundamental concept of waveform-dependent metasurfaces. (a) and (b) Time domain responses of (a) a capacitor and (b) an inductor to a rectified signal. (c) Use of a resistor between metasurface patches leads to absorption of either a short or long pulse. (d) One of the measured samples (inductor-based metasurface). Each circuit element was soldered and electrically connected to the surface. (e) Metasurfaces were built on a 1.52 mm height dielectric substrate (Rogers 3003) and simulated in a TEM waveguide (22 mm tall and 18 mm wide). See Supplementary Information for more detail.
