# Supplementary material for: Waveform Selectivity at the Same Frequency
Source: Sci Rep. 2015 Apr 13;5:9639. doi: 10.1038/srep09639 (PMC4394192; doi:10.1038/srep09639)
Supplement: Supplementary Information [file srep09639-s1.pdf]

Supplementary Information

Waveform Selectivity at the Same Frequency

Hiroki Wakatsuchi, Daisuke Anzai, Jeremiah J. Rushton, Fei Gao, Sanghoon Kim, and Daniel F. Sievenpiper

- **Rectification**
- **Modelling**
- **Simulation method**
- **Measurement method**
- **Low power responses**
- **Voltage in a capacitor and current in an inductor**
- **Metasurface with only diodes and resistors**
- **Theoretical performance of PPM Transmission**

Rectification

Microwave diodes rectify incoming signals to a static field in the following manner. Surface waves can be represented by a cosine function $\cos(2\pi ft)$, where f and t are respectively the frequency and time. Note that for simplicity other variables including the spatial position, phase delay, magnitude, etc, are all omitted here. When the input signal is fourier-transformed (i.e. $\int_{-\infty}^{\infty} \cos(2\pi ft) e^{-j2\pi t} dt$), the output spectrum contains only f.

Table 1: Fourier component of various modes for half and full wave rectifications

Fourier term	W/O rectification: cos	Half wave rectification: (cos+| cos |)/2	Full wave rectification: | cos |
0	0	$1/\pi$	2π
f	1/2	1/4	0
$2f$	0	$1/(3\pi)$	$2/(3\pi)$
$3f$	0	0	0
$4f$	0	$-1/(15\pi)$	$-2/(15\pi)$

If the surface current is rectified by a diode, however, the rectified signal becomes $(\cos(2\pi ft) + |\cos(2\pi ft)|)/2$, which through a fourier transform results in an infinite set of frequencies with decreasing magnitudes. The largest term is at zero frequency, or a static field.

This rectification to a static field is further enhanced, if a full wave rectification is introduced as the metasurfaces demonstrated in the paper. In this case the incoming signal is rectified to $|\cos(2\pi ft)|$. All of these are summarised in Table 1.

Note that full wave rectification is achievable with the metasurface designs used in our study. In our structures an edge of each metasurface patch is charged either positively or negatively. In Fig. 1(c), for example, a current flows into the capacitor/inductor from the left or right patch. However, in either case still the current flow within the four diodes is only one direction (i.e. downward in this figure).

Modelling

Waveform-selective metasurfaces were modelled as described in Fig. 7. Diodes were modelled by a SPICE model whose parameters are summarised in Table 2. The dielectric substrate used (Rogers3003) had relative permittivity of 3.0 and dielectric loss tangent of 0.0013. The relative permeability was 1.0. The conducting patches of the metasurfaces were modelled by 17-μm-thick copper, which had bulk conductivity of $5.8 \cdot 10^7$ S/m, relative permittivity of 1.0 and relative permeability of 0.999991.

Table 2: SPICE model parameters used for diode modelling

Parameter	Units	Value
B_V	V	7.0
C_{J0}	pF	0.18
E_G	eV	0.69
I_{BV}	A	$1 \cdot 10^{-5}$
I_S	A	$5 \cdot 10^{-8}$
N		1.08
R_S	Ω	6.0
P_B (VJ)	V	0.65
P_T (XTI)		2
M		0.5

Simulation method

All the simulations were performed by electromagnetic/circuit co-simulation to evaluate the absorptance A, reflectance R and transmittance T of metasurfaces. First, as drawn in Fig. 1(e), we calculated the scattering parameters through an electromagnetic simulator Ansys HFSS 15.0. The metasurface simulated here had lumped ports which were later connected to circuit components, such as diodes, in circuit simulations performed by a circuit simulator Ansoft Designer 8.0. Effectively, this is equivalent to directly connecting them to the metasurface in the electromagnetic simulation. Hence, all the scattering parameters and absorptance were calculated by the circuit simulator.

The absorbing performances in high power pulse and CW simulations were both evaluated in the time domain using the electric circuit shown in Fig. 8, where for simplicity we abbreviated lumped ports. In these simulations the input power was theoretically estimated by

$$P_{in}(t) = 2P_0 \sin^2(2\pi f t), \tag{6}$$

where P_{in} and P_0 are, respectively, the instant input power and the magnitude. Since the voltage and current meters next to the input port read power P_{m_1} containing both the incident and reflected powers, the reflected power P_{ref} was calculated by subtracting eq. (6) from P_{m_1} (i.e. $P_{ref} = P_{in} - P_{m1}$). For the transmitted power P_{tra}, the meters next to the output port were simply used (i.e. $P_{tra} = P_{m2}$), since there is no signal from the output port.

For the pulse simulations, a switching system was inserted between the input port and neighboring meters as Fig. 9. This system played a role in switching the connection between the metasurface and input port. First, the metasurface model was connected to the original input port which generated a CW signal. Then, the metasurface model was disconnected from the original port and connected to a new port, which did not have any signal source. As a result, an arbitrary width of pulse was produced. The reflected and transmitted energies were then integrated over time and divided by the input energy to

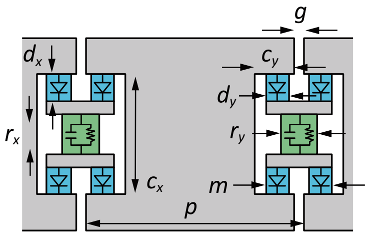

Figure 7: The geometry simulated. The dimensions were given from $c_x = 7.6$, $c_y = 1.7$, $d_x = 1.3$, $d_y = 0.5$, $g = 1.0$, $m = 2.4$, $p = 18.0$, $r_x = 1.0$ and $r_y = 2.0$ (all in mm).

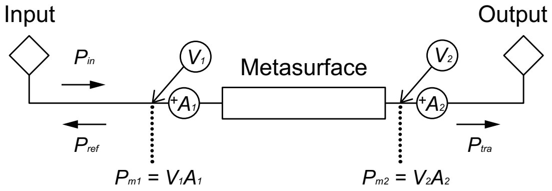

Figure 8: Entire circuit configuration used for the circuit simulation. For the actual model we also connected lumped ports to the metasurface.

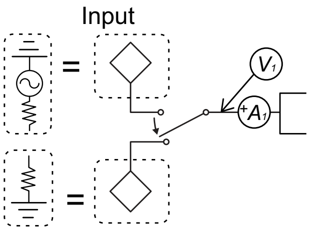

Figure 9: Introduction of switching system to produce pulses. In an arbitrary time the metasurface model was disconnected from the original input port and connected to a new port which did not have any excitation source. Each port had the same impedance as the port impedance of the metasurface model.

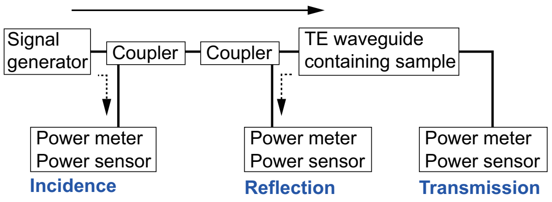

Figure 10: Measurement system.

obtain the reflectance R and transmittance T. These values were subtracted from one to calculate the absorptance A (i.e. $A = 1 - R - T$).

The high power CW simulations were more straightforward, i.e. the simulation was performed until a steady state, where the reflected and transmitted power were averaged and divided by the input power to obtain R and T. There were a couple of factors to lead to instability in the simulations, for example, due to passivity and use of commercial diode models. For this reason some simulations were truncated earlier than others to avoid diverged results.

Measurement method

High power measurements were performed using the measurement system sketched in Fig. 10. An input signal was generated from a signal generator (Agilent N5181A) and passed through waveguides, where metasurfaces were deployed. In addition, directional couplers (KRYTAR 102008010) were used to monitor the incident and reflected signals, which were measured by power sensors (Agilent N1921A). Another set of a power meter and power sensor was used after the waveguides to measure the transmitted power. In order to measure the scattering parameters as accurately as possible, the high power measurements were performed after several calibration steps. Some of the measurement devices were automatically controlled through LabVIEW software such that the input frequency could be quickly swept, which facilitated the measurement process substantially. The pulse width and duty cycle were changed with the signal generator and LabVIEW.

Extra care was used on the pulse measurements. Power meters used (Agilent N1911A) had multiple operating modes, e.g. averaging mode and peak mode. Since our simulation results showed the same peaks for different pulse widths in time domain, we adopted the averaging mode. However, this indicates that depending on the duty cycle of the pulse, we needed to offset the received signals, e.g. if the received signal was 0 dBm and the duty cycle was 10 % (i.e. -10 dB), then the actual magnitude was 10 dBm (i.e. 0 dBm - (-10 dB)). Due to the noise floor of the power meters, the duty cycle could not be set too small, otherwise the measured value would read the noise floor. This issue, however, needed to be compromised with other problems, e.g. the discharging time of stored energy in capacitors. Since the signal generator repeatedly produced pulses, the duty cycle was set long enough to ensure fully discharging the electric charges stored in the capacitors. Because of these two issues we decided to set the duty cycle between 0.1 % and 1 % after some test measurements.

Another point to note here is that the reflection was significantly small in the pulse measurements. Hence, the absorptance was estimated from the transmittance only, i.e. $A = 1 - T$. This fact can be numerically confirmed from Figs. 2(c), 2(f), 3(c) and 3(f) as well, where the reflected powers of all the waveform-selective metasurfaces were very limited, compared to the transmitted powers.

Low power responses

Low power scattering profiles of the capacitor-based metasurface are shown in Fig. 11 [19]. Since the input power was not large enough to turn on the diodes, the metasurfaces behaved as normal conducting surfaces and transmitted the most energy, especially up to 4.2 GHz in the simulation and up to 4.0 GHz in the measurement. The differences can be attributed to parasitics in the circuit elements, especially in the diodes, which were not fully modelled in the simulation for the sake of simplicity.

Since the low power responses do not depend on what components are used within the

diode bridges, the simulation result remains the same even if pairs of a parallel capacitor and resistor are replaced with pairs of a series inductor and resistor as an inductor-based metasurface. This is demonstrated in Fig. 12, which numerically shows the low power scattering profile of the inductor-based metasurface simulated in Fig. 2(d).

Voltage in a capacitor and current in an inductor

In Figs. 1(a) and 1(b) we explained how a capacitor and inductor respond to a rectified incoming wave in time domain. Such behaviours are numerically demonstrated in Figs. 13(a) and 13(b). These figures respectively plot the voltage across the second capacitor of the capacitor-based metasurface used in Fig. 2(b) and the current flowing into the second inductor of the inductor-based metasurface used in Fig. 2(e). As seen in these figures, the capacitor used in the capacitor-based metasurface is gradually charged up, while the inductor-based metasurface allows more current to come in, since the electromotive force is disappearing.

Metasurface with only diodes and resistors

Capacitors and inductors play very important roles to create the waveform selectivity. This is confirmed from Fig. 14, which shows the pulse width dependence and time domain response of the metasurface used in Fig. 2(b) but without the capacitors, namely with only diodes and resistors. Under this circumstance the metasurface no longer exhibits any waveform dependence, because there is no circuit element to temporarily control the incoming energy.

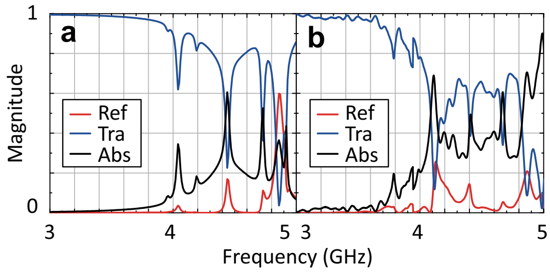

Figure 11: Low power scattering profile of the capacitor-based metasurface in (a) simulation and (b) measurement.

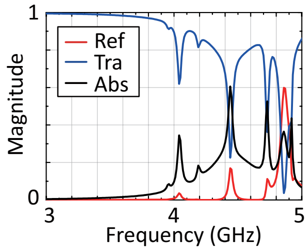

Figure 12: Low power scattering profile of the inductor-based metasurface in simulation.

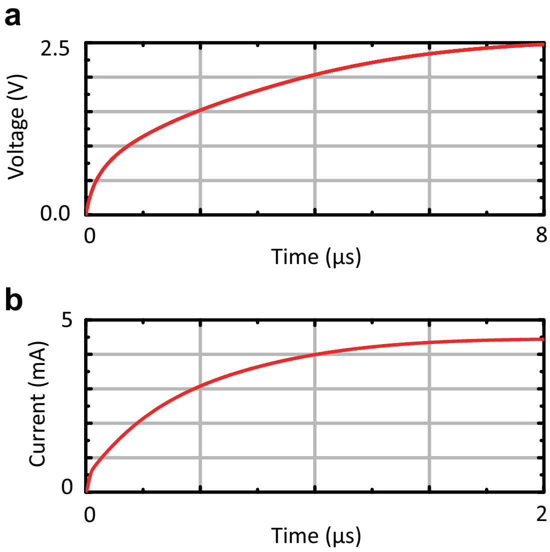

Figure 13: Voltage and current in metasurfaces. The capacitor-based metasurface simulated in Fig. 2(b) gradually increases the voltages across the capacitors as in (a), which plots the voltage of the second capacitor from the front. On the other hand, the inductor-based metasurface simulated in Fig. 2(e) permits more current to come in as (b), which represents the current flowing into the second inductor.

Theoretical performance of PPM transmission

This section explains the theoretical wireless communication performance, i.e. bit error rate (BER) performance, for the PPM transmission under the additive white Gaussian (AWGN) channel. The average BER for the binary PPM transmission with the energy detection can be theoretically analysed as

$$P_b(E_b/N_0) = \frac{1}{2}\exp\left(-\frac{E_b}{2N_0}\right) \tag{7}$$

where E_b/N_0 indicates the energy per bit to the noise power spectrum density ratio. Fig. 15 shows the theoretical BER performance analysed from the above equation. Fur-

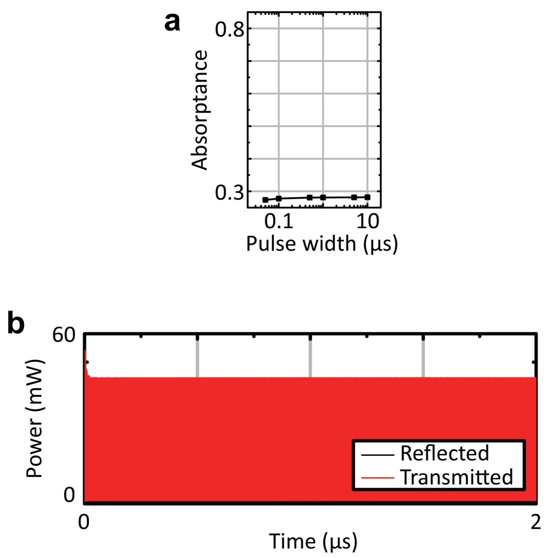

Figure 14: (a) Pulse width dependence and (b) time-domain response of the metasurface used in Fig. 2(b) but without capacitors, namely with only diodes and resistors.

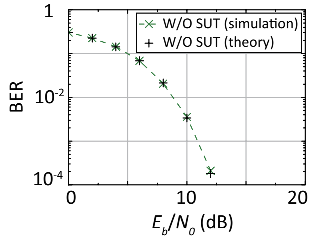

Figure 15: Theoretical analysis and simulation result for the PPM transmission with the energy detection under the AWGN channel.

thermore, this figure also includes the result evaluated from the computer simulation without any metasurface (i.e. just an empty TEM waveguide). As seen in this figure, these two results agree with each other very well. This means that the computer simulation for the wireless communication performance evaluation has been properly performed, and the overall BER results in this paper have a certain level of reliability.
